# Demoralization in acute coronary syndrome: Treatment and predictive factors associated with its persistence^☆^

**DOI:** 10.1016/j.ijchp.2024.100444

**Published:** 2024-01-27

**Authors:** Sara Gostoli, Regina Subach, Francesco Guolo, Sara Buzzichelli, Giovanni Abbate Daga, John M. de Figueiredo, Chiara Rafanelli

**Affiliations:** a Department of Psychology “Renzo Canestrari”, University of Bologna, Viale Berti Pichat 5, 40127 Bologna, Italy; b Division of Cardiology, Bellaria Hospital, AUSL Bologna, Bologna, Italy; c Eating Disorders Center for Treatment and Research, Department of Neuroscience, University of Turin, Turin, Italy; d Department of Psychiatry, Yale University School of Medicine, New Haven, CT, United States

**Keywords:** Acute coronary syndrome, Cognitive-behavioral therapy, Demoralization, Diagnostic criteria for psychosomatic research, Well-being therapy

## Abstract

**Background/objective:**

Although demoralization is associated with morbidity and mortality in cardiac settings, its treatment has been overlooked. The present randomized controlled trial aimed at 1) evaluating the effectiveness of sequential combination of Cognitive-Behavioral and Well-Being therapies (CBT/WBT), compared to Clinical Management (CM), on demoralization among Acute Coronary Syndromes (ACS) patients, at post-treatment and after 3 months; 2) examining ACS patients’ characteristics predicting demoralization persistence at 3-month follow-up.

**Method:**

91 demoralized ACS patients were randomized to CBT/WBT (*N* = 47) or CM (*N* = 44). Demoralization was assessed with an interview on Diagnostic Criteria for Psychosomatics Research at baseline, post-treatment and 3-month follow-up. Predictors of demoralization maintenance included cardiac parameters, psychological distress and well-being.

**Results:**

Compared to CM, CBT/WBT significantly reduced demoralization post-treatment. Somatization (odds ratio = 1.11; *p* = 0.027) and history of depression (odds ratio = 5.16; *p* = 0.004) were risk factors associated with demoralization persistence at follow-up, whereas positive relationships (odds ratio = 0.94; *p* = 0.005) represented protective factors.

**Conclusions:**

The study provides preliminary and promising evidence on the benefits of CBT/WBT in treating demoralization in ACS patients. Moreover, ACS patients with somatization or positive history of depression could be at higher risk for developing persistent demoralization.

Demoralization is a complex transdiagnostic construct expressed by patients as a cluster of symptoms such as subjective incompetence, distress and inability to cope with a stressful situation (de Figueiredo, 1993). Demoralization has been operationally defined according to Diagnostic Criteria for Psychosomatic Research (DCPR; Fava et al., 1995; Rafanelli et al., 2003) to describe feelings of helplessness, hopelessness, and giving-up frequently observed in medical settings (Gan et al., 2022; Rafanelli et al., 2003). It is highly prevalent among cardiac patients (Gan et al., 2022), twice as common as depression (Bailey et al., 2020; Hsu et al., 2022; Rafanelli et al., 2020), and it is associated with low quality of life and adverse clinical outcomes (Hsu et al., 2022), such as poor therapeutic adherence, increased hospitalizations, and higher mortality rates (Rafanelli et al., 2020). To the best of our knowledge, specific literature on demoralization prevalence in patients with ACS is scarce. Only few studies (Gostoli et al., 2023a; Rafanelli et al., 2005) found a rate of demoralization ranging from 19.6 % to 42.7 % among patients who experienced a first episode of ACS.

Consensus has been emerging that psychotherapy relieves demoralization by addressing the patient's morale, subjective incompetence, sense of self, and well-being (Clarke & Kissane, 2002; de Figueiredo, 2007). The treatment of demoralization, however, has been generally overlooked among patients with cardiac illness, such as Acute Coronary Syndrome (ACS). The TREATED-ACS study (Rafanelli et al., 2020) found that a sequential combination of Cognitive-Behavioral Treatment (CBT) and Well-Being Therapy (WBT), compared to clinical management (CM), was associated with a significant improvement in depressive symptoms in ACS patients with demoralization and/or depression, at the end of the treatment. In both groups, benefits persisted at follow-up, even though the differences between them faded away.

Although a large body of literature has identified predictors of persistence of depression among cardiac patients (Wang et al., 2022), not much is known about factors that predict persistence of demoralization, particularly among ACS patients.

Based on these premises, the present study had two objectives: a) to evaluate the efficacy of CBT/WBT, in comparison to CM, at reducing demoralization among ACS patients at the end of the treatment and at 3-month follow-up; b) to examine the characteristics of ACS patients that predict demoralization persistence at 3-month follow-up.

## Method

### Study procedures and participants

This study describes secondary data analysis from TREATED-ACS study (Rafanelli et al., 2020), a longitudinal multi-center randomized controlled trial (RCT), focused on patients with a first episode of acute myocardial infarction or unstable angina (i.e., ACS) who were enrolled at Cardiology Divisions of Maggiore Hospital (Bologna, Italy) and Molinette Hospital (Torino, Italy). The first 100 consecutive ACS patients with a current diagnosis of major/minor depression or dysthymia according to DSM-IV-TR (American Psychiatric Association, 2000), and/or demoralization according to Diagnostic Criteria for Psychosomatic Research (DCPR) criteria (Fava et al., 1995), were randomly allocated to two different groups: cognitive-behavioral therapy followed by well-being therapy (CBT/WBT) or active clinical management (CM). Exclusion criteria comprised a positive history of bipolar disorder, major depressive disorder with psychotic features, substance abuse/dependence during the previous 12 months, suicide risk, and current use of antidepressants and/or psychotherapy.

For the purposes of the present longitudinal interventional study, only ACS patients who met DCPR criteria for demoralization (*N* = 91) were considered and randomly assigned either to CBT/WBT (*N* = 47) or CM (*N* = 44). Therefore, compared to the TREATED-ACS study (Rafanelli et al., 2020), the findings on treatment effectiveness relate to a more homogeneous population of demoralized ACS patients. Psychological data were collected at three observational points by two clinical psychologists, who were blind to treatment assignment: one month after discharge, before any intervention was implemented (baseline, T1), at the end of the intervention (T2), and 3 months after its conclusion (T3).

TREATED-ACS study was approved by the institutional review board of the ethics committees of Bologna (“Comitato Etico Area Vasta Emilia Centro”) and Turin (“Comitato Etico Interaziendale A.O.U. San Giovanni Battista - A.O. C.T.O. Maria Adelaide”). Written informed consent was secured from all patients, after the procedures had been fully explained to them.

### Intervention

CBT/WBT and CM were performed by the same trained psychotherapists and consisted of 12 weekly, 45-min sessions. The sequential administration of CBT (first 8 sessions) and WBT (final 4 sessions) was based on a written protocol (Fava, 2016). CBT techniques, such as exposure and cognitive restructuring, were used to bring the person out of negative functioning and distress that immediately followed ACS. WBT techniques, involving both cognitive restructuring of thoughts/behaviors interrupting well-being and personalized homework assignments, were used to improve or balance one or more psychological well-being dimensions according to Ryff's conceptual model (i.e., environmental mastery, purpose in life, personal growth, autonomy, self-acceptance, and positive relations with others). Both approaches involved the use of a structured self-monitoring diary and personalized suggestions for lifestyle modifications geared toward cardiovascular health, including treatment adherence.

CM, an active form of control group, entailed the same amount of time and attention from a professional figure than the experimental group, but specific techniques (i.e., exposure, use of diary and cognitive restructuring) were proscribed. Such a form of active control—unlike in previous trials that have used treatment as usual (Reavell et al., 2018)—allows discrimination of specific and nonspecific ingredients of the psychotherapeutic approach. It consists of empathic listening, review of patient's clinical status, providing opportunities for disclosure of distress and worries, and encouragement of treatment adherence.

### Assessment

#### Medical variables

Data on cardiac risk factors (i.e., smoke, hypertension, dyslipidemia, family history of cardiovascular disease, diabetes mellitus, left ventricular ejection fraction <40), medications, and comorbidities were collected from medical records. Data from electrocardiograms, echocardiograms, X-rays, blood pressure and blood studies (cholesterol levels, creatinine, glycosylated hemoglobin, C-reactive protein, and coagulation/fibrinolysis biomarkers) were obtained at intake. Global Registry of Acute Coronary Events (GRACE; Tang et al., 2007) risk index was calculated during ACS hospital admission to determine risk of morbidity and mortality both during hospitalization at 6-month post-discharge.

#### Psychological variables

Psychological assessment included both observer- and self-rated measures.

Semi-Structured Interview based on the DCPR (SSI-DCPR; Rafanelli et al., 2003) was administered to assess the presence of demoralization. It has shown excellent inter-rater reliability, with *κ* values ranging from 0.69 to 0.97 (Galeazzi et al., 2004).

Structured Clinical Interview for DSM-IV-TR, Axis I Disorders (First et al., 2002), was used to establish the presence of past or current major/minor depression and dysthymia.

Twenty-item change version of Clinical Interview for Depression (CID-20; Paykel, 1985; Guidi et al., 2010) was used to perform a comprehensive assessment of affective symptoms. It contains 20 items rated on a 7-point Likert scale, with anchor point specified on the severity, frequency, and/or quality of the symptoms. The higher the score, the worse the mood disturbance. CID has been shown to be a sensitive assessment tool in clinical trials, with high inter-rater reliability supported by mean correlations from 0.81 to 0.82 (Guidi et al., 2010).

Symptom Questionnaire (SQ; Benasi et al., 2020; Kellner, 1987), a 92-item self-report questionnaire, was used to assess 4 self-perceived symptoms of depression, anxiety, hostility-irritability, and somatization. The higher the score, the higher the psychological distress. The SQ is a highly sensitive clinimetric index that is recommended to use in clinical trials with correlation coefficients ranging from 0.39 to 0.93 (Benasi et al., 2020).

Psychological Well-Being scales (PWBs; Ruini et al., 2003; Ryff, 1989), an 84-item questionnaire, were used to evaluate 6 psychological well-being dimensions (autonomy, environmental mastery, personal growth, positive relationships, purpose in life, and self-acceptance). Higher scores correspond to greater psychological well-being. Test-retest Pearson's coefficients were satisfactory for all the six scales, especially for personal growth, positive relations, purpose in life and self-acceptance (ranging from 0.78 to 0.82), and the questionnaire showed acceptable validity and reliability across different samples (Ruini et al., 2003).

The scale of stress of the PsychoSocial Index (Piolanti et al., 2016; Sonino & Fava, 1998) was employed to establish the presence of acute or chronic stressful life events. The scale has high interrater reliability, with intraclass correlation coefficients of 0.88 (Piolanti et al., 2016).

### Statistical analysis

Data were analyzed using STATA/IC-64 16.0. The quality of data collection was monitored regularly to assure accuracy and completeness. Given that the present investigation represents a secondary analysis of the TREATED-ACS Study (Rafanelli et al., 2020), the sample size was derived from the main RCT and was determined by including only ACS patients with demoralization. Missing data were handled by complete case analysis, namely only the cases with complete data were analyzed, whereas individuals with missing data on any of the included variables were dropped from the analyses (Dettori et al., 2018).

#### Descriptive statistics

Descriptive statistics were run to study the differences between treatment and control groups with regard to the reduction of the cases of demoralization at T2 and T3. *T*-test statistics were used to study means differences and Chi-square to test differences in frequencies and independence of the categorical variables (first objective of the study).

#### Logistic regression models

ACS patients characteristics predicting demoralization persistence at T3 (second objective of the study) were examined by conducting univariate and multivariate logistic regressions.

#### Univariate regression models

Univariate logistic regression models were used to identify associations between presence of demoralization at T3 (dependent variable) and specific covariates (i.e., treatment group, biomarkers, psychological distress and well-being) without considering the effect of other variables.

#### Multivariate regression models

Multivariate logistic regression models were used to control for all other predictors. Specific lifestyle-related variables documented in the literature, such as smoke, insomnia, and hypercholesterolemia, as well as a specific variable that has been found to be related to demoralization, environmental reactivity (Gostoli et al., 2023a), were included in the analysis. A backward selection procedure including only those variables found to be significantly associated with the dependent variable (presence of demoralization at T3), was performed.

#### Evaluation of model performance

Values of accuracy, sensitivity and specificity of the multivariate model were computed, and model performance was evaluated with the Receiver Operator Characteristic (ROC) curve.

## Results

### Baseline profile of the sample and descriptive statistics

The profile of the sample at T1 and descriptive statistics are presented in Table 1. The mean age of the sample was 58.3 years (SD = 10.62, range 40–84). The majority of the patients were men (68.1 %), married (68.1 %), employed (58.3 %), and high school graduates (44 %).

**Table 1:** Baseline sociodemographic, medical, and psychological profile of the sample.

Variables	CBT/WBT group (*N* = 47)	CM group (*N* = 44)	Total sample (*N* = 91)
Age (years), *mean (SD)*	57.36 (10.01)	59.39 (11.26)	58.34 (10.62)
Sex, *n (%)*			
Males	29 (61.7)	33 (75)	62 (68.13)
Females	18 (38.3)	11 (25)	29 (31.87)
Marital status, *n (%)*			
Single	4 (8.51)	6 (13.64)	10 (10.99)
Married	31 (65.96)	31 (70.45)	62 (68.13)
Separated	4 (8.51)	4 (9.09)	8 (8.79)
Divorced	2 (4.26)	1 (2.27)	3 (3.3)
Widow/widower	6 (12.77)	2 (4.55)	8 (8.79)
Occupation, *n (%)*			
Employed	32 (68.09)	21 (47.73)	53 (58.25)
Unemployed	1 (2.13)	4 (9.09)	5 (5.49)
Retired	12 (25.53)	16 (36.36)	28 (30.77)
Homemaker	2 (4.26)	3 (6.82)	5 (5.49)
Education, *n (%)*			
Primary school	5 (10.64)	4 (9.09)	9 (9.89)
Middle school	14 (29.79)	16 (36.37)	30 (32.97)
High school	18 (38.3)	22 (50)	40 (43.95)
University	8 (17.02)	1 (2.27)	9 (9.89)
Postgraduate education	2 (4.25)	1 (2.27)	3 (3.3)
Type of ACS, *n (%)*			
STEMI acute myocardial infarction	31 (65.96)	28 (63.64)	59 (64.84)
NSTEMI acute myocardial infarction	13 (27.66)	12 (27.27)	25 (27.47)
Unstable angina	3 (6.38)	4 (9.09)	7 (7.69)
Medical procedure for ACS, *n (%)*			
Single PTCA	36 (76.6)	35 (79.54)	71 (78.02)
PTCA with 2+ stents	8 (17.02)	7 (15.91)	15 (16.49)
None	3 (6.38)	2 (4.55)	5 (5.49)
Drug-eluting stent	23 (52.27)	17 (40.48)	40 (46.51)
Cardiovascular risk factors, *n (%)*			
Dyslipidemia/hypercholesterolemia	29 (61.7)	23 (52.27)	52 (57.14)
Hypertension	24 (51.06)	21 (47.73)	45 (49.45)
Smoker (current)	21 (44.68)	19 (43.18)	40 (43.96)
Familiarity	16 (34.04)	11 (25)	27 (29.67)
Diabetes	9 (19.15)	8 (18.18)	17 (18.68)
LVEF < 40	4 (8.51)	2 (4.55)	6 (6.59)
GRACE risk index at admission (mortality), *mean (SD)*			
In-hospital risk, %	3.58 (8.84)	4.36 (8.15)	3.96 (8.47)
6-months risk, %	6.63 (11.95)	8.28 (10.74)	7.43 (11.35)
GRACE risk index at admission (mortality + AMI), *mean (SD)*			
In-hospital risk, %	15.45 (10.15)	16.02 (10.9)	15.73 (10.47)
6-months risk, %	25.06 (13.07)	26.68 (15.46)	25.85 (14.22)
Medications, *n (%)*			
Cholesterol reducers	46 (97.87)	41 (93.18)	87 (95.6)
*β*-blockers	45 (95.74)	44 (100)	89 (97.8)
Platelet aggregation inhibitors	45 (95.74)	42 (95.45)	87 (95.6)
Cardioaspirin	44 (93.62)	42 (95.45)	86 (94.51)
Vasodilators	34 (72.34)	30 (68.18)	64 (70.33)
Angiotensin-converting enzyme inhibitors	28 (59.57)	31 (70.45)	59 (64.84)
Polyunsaturated fatty acids—omega-3	10 (21.28)	10 (22.73)	20 (21.98)
Antihyperglycemics	6 (12.77)	7 (15.91)	13 (14.29)
Diuretics	6 (12.77)	4 (9.09)	10 (10.99)
Angiotensin receptor blockers	5 (10.64)	3 (6.82)	8 (8.79)
Calcium channel blockers	1 (2.13)*	6 (13.64)*	7 (7.69)
α-adrenergic receptor inhibitors	0 (0)	3 (6.82)	3 (3.3)
Antihyperuricemics	0 (0)	2 (4.55)	2 (2.2)
Antiarrhythmic	0 (0)	0 (0)	0 (0)
Heart rate reducers	0 (0)	1 (2.27)	1 (1.1)
7+ medications	10 (21.28)*	20 (45.45)*	30 (32.97)
Medical comorbidities, *n (%)*			
Digestive system diseases	18 (38.3)	20 (45.45)	38 (41.76)
Endocrine diseases	8 (17.02)	5 (11.36)	13 (14.29)
Circulatory/cardiac comorbidities	2 (4.26)	4 (9.09)	6 (6.59)
Prostatic and male reproductive system diseases	3 (6.38)	1 (2.27)	4 (4.4)
Urinary system diseases	2 (4.26)	1 (2.27)	3 (3.3)
Orthopedic diseases	1 (2.13)	2 (4.55)	3 (3.3)
Asthma	3 (6.38)	1 (2.27)	4 (4.4)
Chronic obstructive pulmonary disease	2 (4.26)	1 (2.27)	3 (3.3)
Stroke/aneurysm	1 (2.13)	1 (2.27)	2 (2.2)
Heteroplasia/neoplasia	1 (2.13)	1 (2.27)	2 (2.2)
Hyperuricemia	0 (0)	3 (6.82)	3 (3.3)
Glaucoma	1 (2.13)	0 (0)	1 (1.1)
Multiple sclerosis	0 (0)	0 (0)	0 (0)
Cluster headache	1 (2.13)	0 (0)	1 (1.1)
Cushing disease	1 (2.13)	0 (0)	1 (1.1)
Sarcoidosis	1 (2.13)	0 (0)	1 (1.1)
Thalassemia	0 (0)	1 (2.27)	1 (1.1)
Rheumathoid arthritis	0 (0)	1 (2.27)	1 (1.1)
2+ medical comorbidities	10 (21.28)	11 (25)	21 (23.08)
Mean biomarkers, *mean (SD)*			
Hemoglobin, g/dL	13.91 (1.25)	13.90 (1.34)	13.91 (1.29)
Platelets, *n* x 10^3^/mm^3^	238.91 (56.49)	230.98 (52.09)	235.17 (54.3)
Creatinine, mg/dL	0.94 (0.17)	0.95 (0.21)	0.94 (0.19)
Triglycerides, mg/dL	118.70 (53.39)	125.70 (60.38)	122.04 (56.63)
HDL cholesterol, mg/dL	52.15 (16.95)	46.21 (12.01)	49.31 (15.01)
LDL cholesterol, mg/dL	88.94 (24.81)	94.60 (30.27)	91.64 (27.54)
Total cholesterol, mg/dL	158.53 (30.22)	162.70 (38.84)	160.52 (34.47)
Glycated hemoglobin, mmol/mol	41.15 (8.62)	43.04 (10.26)	42.02 (9.4)
Fibrinogen, mg/dL	350.77 (63.63)	357.80 (70.88)	354.05 (66.81)
D-dimer, mg/dL FEU	0.62 (1.4)	0.47 (0.41)	0.55 (1.06)
HRV^a^, ms	51.21 (28.42)	39.25 (7.27)	45.23 (21.34)
C-reactive protein			
BO, mg/dL	0.19 (0.21)	0.43 (0.74)	0.30 (0.54)
TO, mg/L	0.30 (0.39)	0.67 (1.18)	0.48 (0.88)
Symptom Questionnaire, *mean (SD)*			
Anxiety	8.94 (4.65)	7.45 (4.73)	8.22 (4.72)
Depression	8.02 (4.83)	7.11 (4.62)	7.58 (4.72)
Somatization	9.85 (5.81)	7.95 (5.3)	8.93 (5.62)
Hostility	4.85 (4.07)	5.20 (4.28)	5.02 (4.16)
Psychological Well-Being scales, *mean (SD)*			
Autonomy	62.38 (9.4)	60.98 (9.19)	61.70 (9.28)
Environmental mastery	55.57 (11.77)	54.80 (9.65)	55.20 (10.75)
Personal growth	61.06 (9.69)*	56.59 (10.04)*	58.90 (10.06)
Positive relationships	61.53 (13.61)	59.95 (10.31)	60.77 (12.09)
Purpose in life	56.81 (11.74)	56.05 (9.73)	56.44 (10.76)
Self-acceptance	54.45 (11.85)	55.41 (12.31)	54.91 (12.02)
CID-20 total score, *mean (SD)*	38.17 (8.54)	36.11 (9.12)	37.18 (8.84)
Depression (DSM), *n (%)*	32 (68.09)*	21 (47.73)*	53 (58.24)
Major depression	2 (4.26)	3 (6.82)	5 (5.49)
Minor depression	29 (61.7)*	18 (40.91)*	47 (51.65)
Dysthymia	1 (2.13)	0 (0)	1 (1.1)
History of depression (DSM), *n (%)*	33 (70.21)*	22 (50)*	55 (60.44)
History of demoralization (DCPR), *n (%)*	35 (74.47)	31 (70.45)	66 (72.53)
Chronicity of depression/demoralization, *n (%)*			
Current + previous episode of depression	25 (53.19)	15 (34.09)	40 (43.96)
Current + previous episode of demoralization	35 (74.47)	31 (70.45)	66 (72.53)
High values for CID-20 items, *n (%)*			
Depressed mood	35 (74.47)	29 (65.91)	64 (70.33)
Environmental reactivity	33 (70.21)	35 (79.55)	68 (74.73)
Guilt	27 (57.45)	21 (47.73)	48 (52.75)
Pessimism	30 (63.83)	21 (47.73)	51 (56.04)
Suicidal tendencies	3 (6.38)	4 (9.09)	7 (7.69)
Work and interests	23 (48.94)	17 (38.64)	40 (43.96)
Energy and fatigue	30 (63.83)	23 (52.27)	53 (58.24)
Generalized anxiety	22 (46.81)	21 (47.73)	43 (47.25)
Panic attacks	2 (4.26)	0 (0)	2 (2.2)
Phobic anxiety	16 (34.04)	13 (29.55)	29 (31.87)
Phobic avoidance	11 (23.4)	9 (20.45)	20 (21.98)
Somatic anxiety	17 (36.17)	19 (43.18)	36 (39.56)
Anorexia	7 (14.89)	6 (13.64)	13 (14.29)
Increased appetite	3 (6.38)	6 (13.64)	9 (9.89)
Irritability	20 (42.55)	18 (40.91)	38 (41.76)
Early insomnia	9 (19.15)	3 (6.82)	12 (13.19)
Delayed insomnia	4 (8.51)	4 (9.09)	8 (8.79)
Hostility	0 (0)	1 (2.27)	1 (1.1)
Psychomotor retardation	3 (6.38)	4 (9.09)	7 (7.69)
Agitation	4 (8.51)	4 (9.09)	8 (8.79)
Depressed appearance	14 (29.79)	9 (20.45)	23 (25.27)
Stress event, *n (%)*			
1+ stress events	36 (76.6)	33 (75)	69 (75.82)
2+ stress events	31 (65.96)	32 (72.73)	63 (69.23)
Demoralization assessments, *n (%)*			
Post-treatment^b^	15 (33.33)*	25 (62.5)*	40 (47.06)
3-month post-treatment^c^	14 (32.56)	19 (50)	33 (40.74)

*Note:* ACS, acute coronary syndrome; AMI, acute myocardial infarction; CBT, cognitive-behavioral therapy; CID-20, 20-item Clinical Interview for Depression; CM, clinical management; DCPR, Diagnostic Criteria for Psychosomatic Research; GRACE, Global Registry of Acute Coronary Events; HRV, heart rate variability; LVEF, left ventricular ejection fraction; NSTEMI, non-ST-segment elevation myocardial infarction; PTCA, percutaneous transluminal coronary angioplasty; STEMI, ST-segment elevation myocardial infarction; WBT, well-being therapy; BO, Bologna; TO, Torino.

⁎ *p* < 0.05.

a Assessed only in Torino.

b Not assessed for 6 patients (missing values).

c Not assessed for 10 patients (missing values).

Regarding the cardiac profile of the sample, ST-elevation myocardial infarction (STEMI) was the most frequent form of ACS (64.8 %) and almost all patients (94.5 %) underwent percutaneous transluminal coronary angioplasty (78 % with single-stent application, 16.5 % with 2+ stents). Most frequent cardiovascular risk factors were dyslipidemia/hypercholesterolemia (57.1 %), hypertension (49.5 %) and smoke (44 %).

No differences concerning ACS-related aspects or GRACE risk scores were found when comparing the CBT/WBT group with the CM group.

CM group was prescribed a higher number of medications than the CBT/WBT group ($\chi_{1}^{2}=6.01$; *p* = 0.014). Medications most frequently prescribed at discharge were *β*-blockers (97.8 %), statins (95.6 %) and platelet aggregation inhibitors (95.6 %). Patients in CM group were prescribed significantly more frequently calcium channel blockers ($\chi_{1}^{2}=4.24$; *p* = 0.040) when compared with the patients in CBT/WBT group.

The most frequent medical comorbidities were gastrointestinal (41.8 %) and endocrine diseases (14.3 %). As for comorbid medical diagnoses and levels of biomarkers assessed at baseline, the two groups did not show any significant difference.

Regarding psychological variables, 51.7 % of the patients presented with comorbid minor depression in addition to demoralization. PWB “personal growth” scores (*t*_89_ = 2.16; *p* = 0.033) and frequency of current DSM major depression ($\chi_{1}^{2}=3.87$; *p* = 0.049), DSM minor depression ($\chi_{1}^{2}=3.93$; *p* = 0.047), and positive history of DSM depression ($\chi_{1}^{2}=3.88$; *p* = 0.049), were significantly higher in CBT/WBT group (Table 1). Differences between the two groups on the other psychological variables were not statistically significant.

Regarding CID-20 items, the patients showed high scores (>3 points) for Environmental Reactivity (74.7 %), Depressed Mood (70.3 %) and Pessimism (56 %) items. However, the two groups did not show any significant difference between each other.

As regards variables related to stressful life events, 69 patients (75.8 %) had experienced at least one event, while 63 (69.2 %) had experienced 2+ events. However, differences between the two treatment groups were not statistically significant.

Ten patients (11 %) did not complete the whole study. Compared to completers, patients lost at follow-up did not show any statistical difference regarding baseline characteristics, except for significantly (*p* < 0.05) higher levels of C-reactive protein and lower scores of PWBs personal growth.

### Post-treatment demoralization

At T2, the CBT/WBT group was associated with a significantly higher decrease in the number of cases of demoralization when compared with the CM group ($\chi_{1}^{2}=7.23$; *p* = 0.007). Indeed, 66.7 % (*N* = 32) of patients in the CBT/WBT group did not report demoralization at T2, compared to 37.5 % of CM group (*N* = 19). However, at T3, the difference in frequency of demoralization between the 2 groups was no longer significant. Both groups had patients who continued to exhibit demoralization at T2 (*N* = 40; 47 %) and T3 (*N* = 33; 40.7 %).

### Predictors of demoralization persistence

#### Univariate analysis

Univariate logistic regression looks at each variable included in the regression without considering the interaction effects with the other variables. Table 2 shows the odds ratios (OR) related to the variables included in the regression for demoralization at T3. There were no statistically significant differences between CM and CBT/WBT treatments among patients who presented with demoralization at T3. Moreover, there were no statistically significant differences for socio-demographic variables as well. However, patients who had previous digestive system diseases had a significantly higher probability to present with demoralization at T3, almost 2.5 times higher when compared with patients without a history of digestive system diseases (OR = 2.58; *p* = 0.044).

**Table 2:** Results of univariate analysis of the logistic regression models for demoralization after three months from the treatment.

Variables	Odds ratio	Standard error	Lower confidence interval	Upper confidence interval	*p*-value
Digestive system diseases	2.580	1.216	1.024	6.500	**0.044**
History of depression (DSM)	4.037	2.078	1.473	11.069	**0.007**
Anxiety (SQ)	1.135	0.058	1.027	1.255	**0.013**
Depression (SQ)	1.135	0.058	1.026	1.254	**0.014**
Somatization (SQ)	1.095	0.048	1.005	1.194	**0.039**
Environmental mastery (PWBs)	0.938	0.023	0.895	0.983	**0.008**
Positive relationships (PWBs)	0.949	0.019	0.912	0.987	**0.010**
Somatic anxiety (CID-20)	3.600	1.739	1.397	9.278	**0.008**
2 or more stressful events	2.948	1.590	1.025	8.484	**0.045**
Total stressful events	1.417	0.199	1.076	1.866	**0.013**

*Note:* CID-20, 20-item Clinical Interview for Depression; DSM, Diagnostic and Statistical Manual of Mental Disorders; PWBs, Psychological Well-Being scales; SQ, Symptom Questionnaire.

A positive history of DSM-IV-TR depression represented a risk factor for the presence of demoralization at T3 (OR = 4.04; *p* = 0.007). Regarding scores on the Symptom Questionnaires, higher levels of Anxiety (OR = 1.14; *p* = 0.013), Depression (OR = 1.13; *p* = 0.014), and Somatization (OR = 1.10; *p* = 0.039) were also risk factors for the persistence of demoralization. Regarding CID-20 items, the item related to Somatic Anxiety (OR = 1.55; *p* = 0.023) was a significant risk factor for the presence of demoralization at T3. For each point of the Likert Scale the probability of being demoralized increased by almost 1.5 times, similar reasoning could be done with the dichotomous variable that considers patients who had high scores for the same CID-20 item (OR = 3.60; *p* = 0.008).

The effect of the presence of one or more stressful events had a significant impact on persistence of demoralization at T3. Significant results were obtained with patients who experienced at least two stressful events: having experienced two or more stressful events was a significant risk factor for persistence of demoralization (OR = 2.95; *p* = 0.045) (Table 2). For each unit increase in the number of experienced stressful events, the probability of demoralization at T3 significantly increased more than 1.4 times (OR = 1.42; *p* = 0.013).

As regards PWB items, patients with higher scores on environmental mastery (OR = 0.94; *p* = 0.008) and positive relationships (OR = 0.95; *p* = 0.010) were less likely to continue demoralized than patients with lower scores on those dimensions. Higher levels on those two PWBs dimensions were potentially significant protective factors (Table 2).

#### Multivariate analysis

Multivariate analysis (backward selection procedure) takes into consideration the effects of other covariates. The effect of digestive system disease, the SQ scores related to Anxiety and Depression, the CID-20 item related to Somatic Anxiety, the PWBs score related to environmental mastery, lifestyle variables, and environmental reactivity lost their statistical significance. On the other hand, SQ somatization kept and increased its strength as significant risk factor for persistence of demoralization (OR = 1.11; *p* = 0.027). Also, a positive history of DSM-IV-TR depression (OR = 5.16; *p* = 0.004) showed a strong association with the outcome and increased the risk of being demoralized 7.2 times after 3 months from the end of the treatment. As regards protective factors, higher scores on PWBs items related to positive relationships significantly reduced the probability of persistent demoralization by almost 7 % for each point of the PWBs score (OR = 0.94; *p* = 0.005) (Table 3).

**Table 3:** Results of multivariate analysis of the logistic regression models for demoralization after three months from the treatment.

Variables	Odds ratio	Standard error	Lower confidence interval	Upper confidence interval	*p*-value
Constant	4.522	6.288	0.296	69.005	0.278
History of depression (DSM)	5.164	2.966	1.675	15.921	**0.004**
Somatization (SQ)	1.114	0.054	1.013	1.225	**0.027**
Positive relationships (PWBs)	0.937	0.022	0.896	0.980	**0.005**

*Note:* DSM, Diagnostic and Statistical Manual of Mental Disorders; PWBs, Psychological Well-Being scales.

#### Model performance

**Fig. 1:**
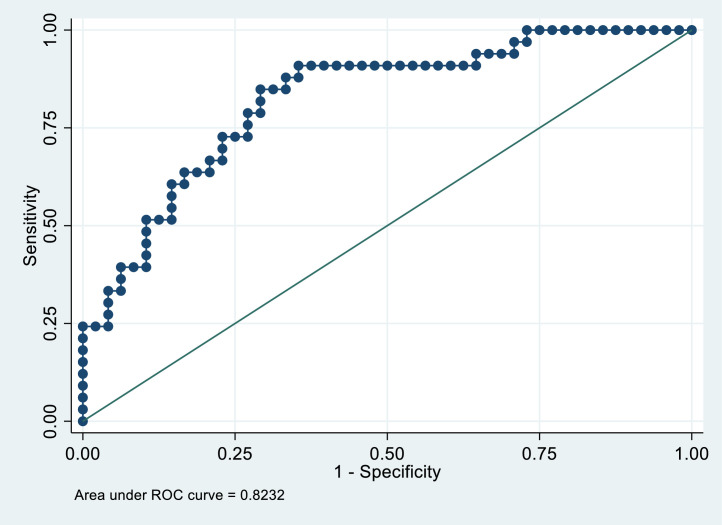
ROC curve of the multivariate model for demoralization after 3 months from intervention.

*Legend:* Sensitivity: percentage of demoralized patients correctly classified; Specificity: percentage of non-demoralized patients correctly classified; ROC curve: Receiver Operating Characteristic curve.

In order to evaluate the performance of the multivariate model, we computed the values of specificity and sensitivity of the model, the Receiver Operating Characteristic (ROC) curve, and the area under the ROC curve (AUC). As regards model performance, the model had good accuracy, with 76.5 % of patients correctly classified with high sensitivity and specificity (84.9 % and 70.8 % respectively) at optimal cut-off point (0.3719). The area under the ROC curve was 0.8232. The high AUC value (> 80 %) shows that the model performance was adequate (Fig. 1).

## Discussion

To our knowledge, this represents the first study addressing the effectiveness of psychotherapeutic interventions in the treatment of demoralization among ACS patients. While factors associated with onset of demoralization among cardiac patients, such as severity of disorder, physical discomfort, changes in lifestyle and living alone (Hsu et al., 2022), have been well described, variables related to the decrease or persistence of demoralization after a targeted psychotherapeutic intervention remain poorly understood, particularly among ACS patients. The results are consistent with the hypothesis that CBT/WBT is more effective and has a faster impact on demoralization among ACS patients at the end of the treatment than CM. It should be noted that CBT/WBT includes specific intervention elements that address not only the characteristics of demoralization (de Figueiredo, 1993; Gan et al., 2022; Rafanelli et al., 2003) such as subjective incompetence, distress and inability to cope with stressful situations, but also factors associated with demoralization, such as psychological well-being, quality of life, and psychological distress (Hsu et al., 2022). Indeed, CBT/WBT is a comprehensive intervention focused on mood disorders (CBT), psychological impairments (WBT) and unhealthy behaviors (lifestyle intervention). Moreover, it appears that specific prescriptions of healthier lifestyle behaviors might be an important aspect to address in order to treat demoralization among these patients (Gostoli et al., 2023b; Rafanelli et al., 2020). However, the difference between the 2 groups tended to fade away over time and CM, as an active form of control group, also showed a positive effect on demoralization decrement at 3-month follow-up. It is likely that non-specific elements of CM intervention, such as offering a healing setting, encouragement, and instilling hopes of improvement, could be beneficial for demoralization reduction among a small group of ACS patients with high susceptibility to these elements. Although both interventions showed positive results in demoralization reduction, 40 % of the sample still experienced demoralization at 3-month follow-up.

The present investigation appears to be the first study that attempts to describe factors associated with persistence of demoralization among ACS patients. Multivariable controlling analysis revealed that somatization and positive history of depression represent risk factors of demoralization persistence among ACS patients at 3-month follow up. Indeed, demoralization, as outlined in DCPR conceptualization, is a widely recognized concept in the field of psychosomatic medicine (Gan et al., 2022). Among cardiac patients in particular, demoralization is associated with various somatic symptoms such as breathing difficulty, fatigue, and pain (Yi-Tsen et al., 2021). Drawing from the evidence in the field, due to the traumatic nature of the ACS and its potential for ongoing physical discomfort and disability, these patients become more aware of potential triggers that might worsen their symptoms (Gostoli et al., 2023b) or increase the risk of future cardiac events (Ganz et al., 2022). Therefore, experiencing somatization might affect their motivation to engage in healthy behaviors and result in increased morbidity and mortality (Ejdemyr et al., 2021).

The present findings highlight the importance of considering the demoralization construct in psychosomatic settings as well as the longitudinal course of mood disorders in the treatment of demoralization in ACS patients. Indeed, a positive history of depression seems to be an ongoing vulnerability not only for subsequent mood disorders (Irwin et al., 2023; Wang et al., 2022), but also for persistence of demoralization (Grassi et al., 2020).

By contrast, ACS patients with positive relationships were less likely to report demoralization at 3-month follow-up than those without positive relationships. Having positive relationships, therefore, could be considered a protective factor against persistent demoralization. Receiving encouragement, reassurance, assistance and engaging in social activities and positive health behaviors (Hsu et al., 2022) are likely to help patients to cope with stress and the challenges related to ACS. Along the same line, but on the reverse side, demoralized ACS patients with fewer positive relationships were less likely to participate in secondary prevention and reported a worse cardiac course (Gostoli et al., 2023b).

The current study presents some limitations that should be noted. The relatively small sample size, the recruitment from only two hospitals in Italy and the nature of the study population itself, which does not fully reflect the “real-world” clinical practice (since an active form of control group - rather than treatment as usual - was included), may impact the generalizability of the results and could potentially have affected the outcomes of interest. Moreover, secondary analyses of RCT data might introduce intrinsic challenges associated with possible confounding variables and biases inherent subsequent evaluations of data that were originally collected for different primary purposes. However, the present findings provide preliminary promising evidence regarding approaches to the treatment of demoralization and possible factors related to its persistence. The findings of this study expand our knowledge on the treatment of demoralization among ACS patients and methods to prevent its persistence.

In conclusion, given that demoralization represents a vulnerability factor for morbidity and mortality in the setting of cardiac disease (Rafanelli et al., 2020), it is important to treat it, taking into consideration the clinical utility of a sequential intervention based on CBT and WBT techniques addressing lifestyle, as well as specific aspects associated with demoralization maintenance or remission (e.g., somatization and positive relationships). Finally, findings of the present study support the literature (Wang et al., 2022) underlining the importance of evaluating the longitudinal course of psychiatric disorders, especially mood disturbances, since the presence of a positive anamnesis for mood disorders could help to identify subgroups of vulnerable patients at higher risk for developing future mental disorders.

## Funding

This work was supported by a grant from “Compagnia di San Paolo di Torino” (Italy) to Chiara Rafanelli M.D., Ph.D., Department of Psychology “Renzo Canestrari”, University of Bologna (Italy).

## Declaration of competing interest

The authors declare that they have no known competing financial interests or personal relationships that could have appeared to influence the work reported in this paper.
